# The Protective Effect of Lipoic Acid on Selected Cardiovascular Diseases Caused by Age-Related Oxidative Stress

**DOI:** 10.1155/2015/313021

**Published:** 2015-04-08

**Authors:** Beata Skibska, Anna Goraca

**Affiliations:** ^1^Department of Applied Pharmacy, Department of Pharmacy, Medical University of Lodz, Muszynskiego 1, 90-151 Lodz, Poland; ^2^Department of Cardiovascular Physiology, Medical University of Lodz, Mazowiecka 6/8, 92-215 Lodz, Poland

## Abstract

Oxidative stress is considered to be the primary cause of many cardiovascular diseases, including endothelial dysfunction in atherosclerosis and ischemic heart disease, hypertension, and heart failure. Oxidative stress increases during the aging process, resulting in either increased reactive oxygen species (ROS) production or decreased antioxidant defense. The increase in the incidence of cardiovascular disease is directly related to age. Aging is also associated with oxidative stress, which in turn leads to accelerated cellular senescence and organ dysfunction. Antioxidants may help lower the incidence of some pathologies of cardiovascular diseases and have antiaging properties. Lipoic acid (LA) is a natural antioxidant which is believed to have a beneficial effect on oxidative stress parameters in relation to diseases of the cardiovascular system.

## 1. Introduction

**Figure 1:**
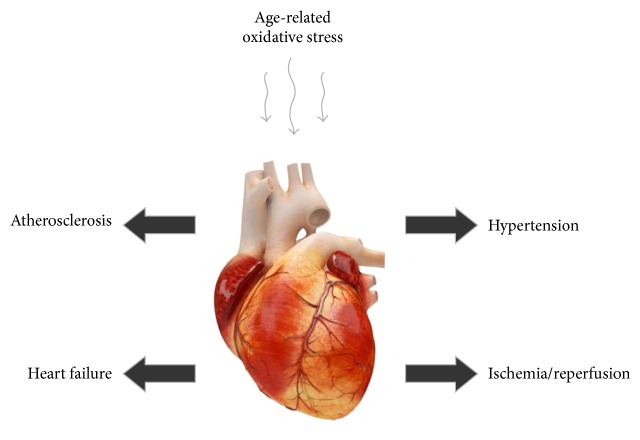
The adverse effect of age-related oxidative stress on some cardiovascular diseases: atherosclerosis, hypertension, ischemia-reperfusion, and heart failure.

Oxidative stress plays a key role in the development of many cardiovascular diseases, including atherosclerosis, hypertension, ischemia-reperfusion injury, and heart failure (Figure 1). There are many factors associated with oxidative stress, which lead to the development of these diseases. One of the main factors is overproduction of ROS, together with decreased nitric oxide bioavailability and reduced antioxidant capacity in the vasculature [1].

Death due to cardiovascular diseases is the cause of mortality in 80% of people aged over 65 years. In addition, the aging process is associated with oxidative stress in the blood vessels and in the heart, which leads to the development of cardiovascular disease (CVD) [2].

According to the free radical theory of aging developed by Harman, the antioxidant defense mechanisms become less effective in people after the age of 40 [3, 4]. This results in fatty acid oxidation and lipid peroxidation, with consequent changes in the physical properties of cell membranes and phospholipids. As they have long half-lives and increased polarity, phospholipid peroxides are active intermediaries of the oxidation and reduction chain [5], which may migrate from point of origin to other places in the organism.

Excessive ROS production and weakened antioxidant mechanisms lead to the occurrence of oxidative stress and induction of apoptosis. ROS reacts with DNA, proteins, and lipids, resulting in the accumulation of products, the onset of degenerative processes, and, ultimately, the development of many serious diseases and aging. Although aging is a natural process, it is accelerated by ROS production.

Oxidative stress is an imbalance between production of ROS present in cells and the biological ability to detoxify the reactive intermediates or repair the harm caused [6]. Currently, antioxidants are used in order to reduce the production of ROS in cells and limit their harmful effects on the organism. One effective antioxidant is lipoic acid (LA).

LA is a natural antioxidant synthesized in the mitochondria of the liver and other tissues [7], which plays a crucial role in metabolism. Its antioxidant properties were first discovered in the 1950s [6] and later confirmed by subsequent studies [8–11]. Its strong reduction and low oxidation-reduction potential (−0.29 V) have made it the subject of many studies from various fields of medicine. It is currently regarded as one of the most potent cellular oxidation regulators [12]. LA is a remarkable compound that appears to slow the process of aging in animal experiments. Considering the strong antioxidant properties of lipoic acid, the purpose of this review is to present the protective role of LA on selected cardiovascular diseases.

## 2. Age-Related Oxidative Stress in Cardiovascular Diseases

### 2.1. Endothelial Dysfunction and Atherosclerosis

Endothelium of the blood vessels is involved in many physiological and pathological processes. It plays a very important role in the physiological regulation of vascular tone, vascular smooth muscle cell migration, cellular adhesion, and resistance to thrombosis [13].

Pathological processes which occur in blood vessels cause the endothelial balance to become dysregulated. This endothelial dysfunction contributes to the development of atherosclerosis, improper blood circulation, inflammation, and even cancer progression [14]. Vascular dysfunction is caused by reduction of nitric oxide levels, production of vasoconstrictor/vasodilator factor imbalances, impaired angiogenesis, endothelial cell senescence, and oxidative stress [15]. Although there are several conditions that contribute to endothelial dysfunction, increased oxidative stress seems to play an important role.

The overproduction of ROS is a result of the adverse effect of oxidative stress on cellular levels of nitric oxide (NO), an important endothelial factor. Recent studies suggest that NO is an important factor for the proper functioning of endothelial cells, because it controls the function of smooth muscle and exerts an antihypertensive effect at the cardiovascular level [16].

NO is synthesized from l-arginine by the enzyme NO synthase (NOS). There are three NOS isoforms: the neuronal isoforms (nNOS), the constitutive endothelial isoform (eNOS), and the inducible isoform (iNOS) [17]. The reduction of NO availability disturbs its vascular homeostasis.

Aging is a physiological process, but it also influences the destabilization of endothelial cells.

This process, and its associated increased oxidative stress, is one of the factors which may cause endothelial dysfunction. The consequence of increased oxidative stress in aging is inactivation of NO by high concentrations of O_2_^•−^ produced by the reaction of NO with ROS [18, 19]. The reaction between NO and O_2_^•−^ forms the peroxynitrite anion (ONOO^−^). This form is known as a reactive nitrogen species (RNS) and characterized high reactivity with proteins, DNA, and lipids. Unlike O_2_^•−^, ONOO^−^ can penetrate into the cardiovascular cells and cause oxidative modifications within them [20]. Peroxynitrite has also been shown to induce microvascular hyperpermeability by disrupting the adherens junction proteins [21].

One* in vitro* study shows decreased eNOS expression in aged human umbilical vein endothelial cells. This process is associated with dysfunction of cell-cell junctions and microvascular hyperpermeability [22]. It leads to severe oxidative injury, which results in cell necrosis or apoptosis. This has been confirmed by many other studies which suggest that the decreased endothelial NO production promotes endothelial cell apoptosis and leads to microvascular rarefaction [23, 24].

Oxidative stress is known to activate redox-sensitive cellular signaling pathways, which have in turn been implicated in inflammation associated with vasculature subjected to aging [25]. According to* in vitro* studies on endothelial cells, this inflammation induces overproduction of ROS and endothelial dysfunction in older rats [26].

In recent years, longevity genes have been identified that affect lifespan and the rate at which the organism ages. For example, defects in mouse Klotho gene have been shown to be associated with endothelial dysfunction, leading to the premature development of atherosclerosis and, at the same time, accelerated aging [27].

Mouse models of mild dyslipidemia have been found to demonstrate endothelial dysfunction, for example, those which are deficient in apolipoprotein E. This endothelial dysfunction is associated with stretch-induced hypercontractility and diminished endothelium-dependent vasorelaxation, accompanied by decreased levels of NO and eNOS, as well as increased plasma levels of IL-6, a proinflammatory cytokine that reduces eNOS levels and activity. Endothelial dysfunction was found to precede the appearance of atherosclerosis in a murine model of dyslipidemia [28].

Atherosclerosis is a pathological state of the vasculature which progresses together with endothelial dysfunction caused by dyslipidemia, leading to the deposition of inflammatory cells and lipids in the vascular wall. Therefore, the state of the aging blood vessels, which are progressively damaged, primarily impacts the development of atherosclerosis. The oxidative stress theory of atherosclerosis indicates that the production of ROS stimulates oxidized low-density lipoprotein formation (ox-LDL) [29].

ox-LDL has many important properties which may promote atherosclerosis. It stimulates vascular ROS formation and causes endothelial dysfunction via NOX activation and endothelial (NO)-synthase (NOS) uncoupling [30]. In addition, Meisinger et al. note that it acts as a proatherogenic marker [31] and that elevated levels of ox-LDL may predict coronary heart disease events in healthy subjects. Moreover, ox-LDL is known to promote oxygen radical generation in human aortic endothelial cells (HEAC) by phosphorylating the p66Shc adaptor protein at Ser36 [14]. Hence, oxidation of LDL appears to contribute to the prooxidant environment in atherosclerotic lesions.

Current research shows that endothelial dysfunction also plays an important role in early and late mechanisms of atherosclerosis development.

Atherosclerosis is known to result in vascular events such as hypertension, ischemic heart failure, and heart failure.

### 2.2. Hypertension

Oxidative stress and aging are involved in hypertension. Both lead to overproduction of reactive oxygen species. The ROS generated in cardiovascular cells cause various forms of pathological vascular damage in blood vessels related to the promotion of cell growth, the accumulation of extracellular matrix protein, inflammation, and endothelial dysfunction, all of which are characteristic features of the hypertensive vascular phenotype [32].

As in atherosclerosis, one of the major mechanisms by which oxidative stress may promote hypertension is endothelial dysfunction. Aside from impaired vascular expansion, the most important effects of endothelial dysfunction are those concerned with two substances produced by the endothelium: NO and endothelin-1 (ET-1) [33]. An imbalance between these substances interferes with vascular homeostasis, leading to vasoconstriction and elevated blood pressure [34]. Disturbed homeostasis is characterized by an increase in the vasoconstriction factor ET-1 and a reduction of the bioavailability of NO [35]. Experimental evidence indicates that NO is inactivated by ROS, particularly O_2_^•−^ and H_2_O_2_, leading to endothelial dysfunction and vasoconstriction [36]. Other studies have shown that ROS can be also generated in response to ET-1 [37].

ET-1 is a vasoconstrictor peptide, which raises blood pressure and induces vascular and myocardial hypertrophy [38]. ET-1 production is known to be influenced by a number of factors. Oxidative stress may cause modulation of ET-1 and in ET-1-induced activation of various signaling pathways [39]. In addition, aging may increase the release of ET-1 from endothelial cells in humans and animals [40, 41]. In turn,* in vitro* studies have shown that ET-1 itself activates many factors, including NF*κ*B and TNF-*α*, which are involved in cell growth, inflammation, and proliferation [42–44]. These processes can also affect the development of hypertension.

Along with NO deficiency and increased ET-1 production, a dysfunctional endothelium also acts as a source of other mediators and factors such as prostaglandin H2, tromboxane A2, ROS and angiotensin II (AT II) that damage vascular cells [45]. Both angiotensin II and endothelin-1 play important roles in age-related endothelial dysfunction [46]. Angiotensin II stimulates ET-1 release and raises blood pressure by a variety of actions [47] and is a potent activator of nicotinamide adenine dinucleotide phosphate (NAD(P)H) oxidase in vascular cells [48]. NAD(P)H is a major source of ROS in the blood vessels and is considered to be a critical determinant of the redox state of blood vessels [49]. Some studies suggest that enhanced NAD(P)H-oxidase activity can be observed in hypertension-induced oxidative stress and subsequently endothelial dysfunction. Another study confirms the role of NAD(P)H-oxidase on the formation of ROS in blood vessels. The activation of NAD(P)H-oxidase in cerebral blood vessels causes H_2_O_2_-mediated opening of BKCa channels in cerebral arteries, leading to consequent hyperpolarization and vasodilation [50]. In addition, oxidative stress activates other enzymes, including mitochondrial enzymes, NOS, and xanthine oxidase, which are produced following ROS production and have a damaging influence on blood vessels.

The relationship between oxidative stress and hypertension has been shown in many experimental models [51–54]. Similarly, the renin-angiotensin aldosterone system (RAAS) is important in the pathogenesis of arterial ageing [55]. RAAS is one of the most important hormonal systems; it oversees the functions of the cardiovascular, renal, and adrenal glands by regulating blood pressure, fluid volume, and sodium and potassium balance. Disorders in RAAS function lead to endothelium dysfunction, which may be caused, inter alia, by age-related oxidative stress [56].

To summarize, hypertension may be triggered by a number of factors. However, oxidative stress and aging both exert a significant influence.

### 2.3. Atherosclerosis-Ischemic Heart Disease

Age-related oxidative stress also leads to cardiac ischemic and reperfusion injuries. Aging and oxidative stress play important roles in the senescent heart. The aged myocardium has less tolerance to ischemia and hemodynamic stress than the young myocardium [57–59].

Many metabolic and biochemical changes in myocardial tissue are the result of oxygen and nutrient deprivation during ischemia. In most cases, the presence of atherosclerotic plaques slowly leads to the narrowing of blood vessels and impairs the blood supply to the heart. Long-term ischemic heart disease can lead to myocardial infarction due to myocardial hypoxia and accumulation of waste metabolites. This can lead to damage to the cardiovascular and cell death by apoptosis [60].

During reperfusion, the concentration of superoxide anions (O_2_^•−^) and hydroxyl groups (OH^−^) from mitochondria is greatly increased. Oxidative stress is then intensified by the increased production of these ROS, which then results in oxidation of mitochondrial proteins and mitochondrial dysfunction [61].

ROS such as superoxide anions, hydrogen peroxide, and hydroxyl groups can cause mitochondrial genomic damage and a gradual decline in mitochondrial function in senescent hearts [62]. Mitochondria from aged hearts have been found to demonstrate reduced membrane potential, which may contribute to lowered adenosine 5′-triphosphate (ATP) synthesis [63]. This imbalance between the synthesis and consumption of ATP significantly influences the metabolism of the heart muscle, leading to greater oxygen consumption. ATP deficiency is also associated with a rapid loss of myocardial contractility, which can result in dysfunctions of the cardiovascular system and arrhythmias [64].

ROS can also activate some biochemical pathways in blood vessels, resulting in changes in cell function. In response to angiotensin II induction, they can activate protein kinase B in vascular smooth muscle cells (VSMC), leading to VSMC hypertrophy [65]. The activation of biochemical signaling pathways promotes greater cellular dysfunction and impairs cardiomyocyte functionality [66].

Increased levels of inflammatory markers such as TNF-*α*, CRP, and IL-6 can be observed in ischemia-reperfusion damage. These compounds and other cytokines can increase the production of ROS in atherosclerosis by stimulating vascular myocytes. Conversely, by inducing inflammation, ROS may also further stimulate the production of inflammatory cytokines.

Furthermore, other biomarkers of oxidative stress play important roles in the pathophysiology of ischemia-reperfusion damage in myocardial infarction. Extracellular biomarkers of ischemia-reperfusion damage include lipid peroxidation products, plasma antioxidant vitamin levels, total antioxidant capacity of plasma, and protein carbonylation. In addition, such intracellular biomarkers as antioxidant enzyme activity, thiol index (GSH/GSSG ratio), carbonyl levels, and F2-isoprostane level can influence the degree of oxidative stress [67].

In general, an imbalance between the demand for oxygen and nutrients and the ability to deliver them to the heart muscle, known as ischemia-reperfusion, is most commonly caused by atherosclerosis, but oxidative stress and related overproduction of ROS also play important roles. They cause lipid, protein, and DNA oxidation, potentially contributing to contractile failure [68].

Ischemia can lead to various diseases of the heart such as heart failure and, ultimately, myocardial infarction, depending on the duration and extent of ischemia.

### 2.4. Heart Failure

The effects of oxidative stress on aging on the vasculature and on the heart muscle are varied but can lead to the development of heart failure (HF). Several cardiovascular diseases are connected with HF, for example, ischemic heart disease, atherosclerosis, hypertension, and cardiac hypertrophy. Oxidative stress and ROS accumulation contribute to all these and contribute to their progression.

In myocardial ischemia, hypoxia and reoxygenation elevate ROS production in cardiac tissues, which leads to direct oxidative damage to cellular components.

ROS influence the function of the extracellular matrix, which is demonstrated by greater interstitial and perivascular fibrosis [69].

On the cellular level, mitochondria are one of the major sites for the generation of ROS, which is an undesirable side product of the energy production. Therefore, mitochondrial dysfunction increases the risk of heart failure. Among the damage induced by ROS generated at the cellular level, mitochondrial DNA (mtDNA) remains the major target [70]. In experimental models, it has been proven that mtDNA deletions contribute to the phenotype of systolic heart failure through increased mtROS [58].

Oxidative stress changes gene expression and influences cell death in heart cells which are now known to exert an influence on heart failure and myocardial remodeling. Heart failure itself is known to involve a decrease in contractility, myocardial fibrosis, myocyte apoptosis, and metabolic remodeling [71]. Metabolic remodeling in heart failure is characterized by decreased cardiac energy production, which is the result of a decrease in the level of ATP in cardiomyocytes. This may lead to progressive impairments in substrate utilization and mitochondrial biogenesis and function. In addition to ATP deficiency, metabolic remodeling involves changes in metabolic pathways that regulate essential, non-ATP-generating cellular processes such as growth, redox homeostasis, and autophagy [72]. A reduced supply of ATP necessary for the contractile function of cardiomyocytes can account for chronic heart failure.

One cause of these processes is increased oxidative stress, which leads to the disruption of the structures of proteins, lipids, and nucleic acids. Several studies have demonstrated an association with these structural disorders and heart failure [73, 74].

In the failing heart, overproduction of ROS leads to the accumulation of superoxide anions, which may be generated by both metabolic and enzymatic sources, including nitric oxide synthase, NADPH oxidases, mitochondrial respiration, and xanthine oxidase [75].

Xanthine oxidase (XO) in particular exerts an important influence on heart failure. XO can also combine with other compounds and enzymes and create reactive oxidants, as well as oxide substrates. It has been found to be present in higher levels with greater activity in cases of heart failure. This upregulation can contribute to the energy disorder in myocardial cells [76].

Similarly, increased NAD(P)H activity has been observed in myocardial cells from humans with heart failure [77, 78]. This increase is due partly to the presence of increased concentrations of angiotensin II, which leads to an imbalance in the oxidative/nitrosative system [27]. In addition, ROS generated by NADPH oxidase proteins are also important in redox signaling [79].

In summary, multiple factors are involved in the etiology of heart failure, and oxidative stress is one of them. To reduce or prevent the adverse effects of oxidative stress on the organism, substances with antioxidant properties can be applied. Research indicates that dietary supplementation by exogenous antioxidants can play a key role in ameliorating many of the effects of oxidative stress in cardiovascular diseases.

## 3. Protective Effect of Lipoic Acid (LA) on Cardiovascular Diseases

Lipoic acid (LA) is a specific antioxidant; it can easily quench radicals, has an amphiphilic character, and does not exhibit any serious side effects [80]. LA a compound that contains sulfur in the form of two thiol groups [81] acts as a cofactor for several mitochondrial enzymes by catalyzing the *α*-ketoacid. The antioxidant properties of LA are based on its ability to directly scavenge ROS, its metal chelating activity, and its potential to react with, and regenerate, other antioxidants such as glutathione and vitamins E and C [82]. LA also demonstrates anti-inflammatory properties.

An additional advantage of LA is its solubility both in water and in fat, which allows it to travel to all parts of the body [83]. Because of its special properties, it is able to enter certain parts of the cell that most other antioxidants are not able to reach.

This compound acts by many mechanisms and can therefore be a very effective antioxidant. Hence, LA is used in various diseases concerning age-dependent oxidative stress. It can be particularly effective in cardiovascular diseases, including ischemic heart disease, hypertension, heart failure, and atherosclerosis, where it may slow aging and prolong lifespan.

### 3.1. Effect of Lipoic Acid in Atherosclerosis

Many studies have confirmed that LA can improve vascular function and decrease the atherosclerotic plaque burden [84, 85]. By chelating redox-active transition metal ions, LA is thought to inhibit the Fenton-like-reaction mechanism and inhibit the formation of OH^−^. As a consequence, lipid peroxidation is inhibited in mitochondria [86].

A crucial regulator of vascular homeostasis is the renin-angiotensin-aldosterone system (RAAS). A key role in the pathogenesis of atherosclerosis is played by angiotensin II (Ang II). It induces oxidative stress and creates superoxide anions primarily through the activation of NAD(P)H-oxidase in vascular cells and myocytes. In addition, Ang II activates intracellular signaling pathways and upregulates many inflammation factors including chemokines, cytokines, and growth factors, which have been implicated in atherosclerotic plaque development.

LA reacts with ROS, such as superoxide anions, normalizes NADPH oxidase activity, and can prevent Ang II-induced macrophage, monocyte, and T cell infiltrations. It is also thought that LA can block AT1 receptors, which improves endothelial function and reduces plaque area in atherosclerosis [87].

Many clinical studies have shown that the beneficial effects of LA against Ang II are linked not only to scavenged ROS, but also to NF-kappaB inhibition. LA reduces NF-*κ*B-mediated inflammatory responses by regulating the expression of proinflammatory genes and adhesion molecules [88]. It also reduces the chemokine and adhesion molecules involved in T cell trafficking to inhibition of monocyte-endothelial interactions by atherosclerotic plaque.

Many animal and human studies report that LA supplementation can result in reduced cholesterol levels [89, 90]. LA may also prevent LDL oxidation by reducing the concentrations of LDL-C, Ox-LDL, serum TC, and lipoprotein (a) [Lp(a)], as well as other oxidative biomarkers [85].

Clinical studies confirm that LA may also reduce the aortic expression of adhesion molecules and the accumulation of aortic macrophages and proinflammatory cytokines, resulting in reduced LDL level and triglyceride concentration and elevated HDL [91, 92]. In animal models, 12-week administration of LA reduced oxidative stress and improved vascular reactivity in animals fed with a high cholesterol diet [93]. LA may also be capable of initiating LDL receptor synthesis in the liver, resulting in increased return of cholesterol to the hepatic system and elevated synthesis of apoprotein A component (a HDL particle moiety) for reversed cholesterol transport [94–96].

Finally, it can be concluded that LA has a direct lipid modulating action and an indirect effect on blood lipid levels, leading to reduced risk of atherosclerosis. It is used as a dietary supplement, either alone or with other oxidants; for example, vitamins C and E may represent a helpful strategy in reducing the adverse effects of oxidative stress.

### 3.2. Effect of Lipoic Acid in Hypertension

Hypertension increases the production of various inflammatory biomarkers. These include chemokines, such as monocyte chemoattractant protein 1 (MCP-1), adhesion molecules, such as P-selectin, and cytokines, such as tumor necrosis factor-*α* (TNF-*α*) and interleukin- (IL-) 6. This elevated production of biomarkers results in reduced NO bioavailability, via NO degradation in vessel cells, and excessive production of endothelin I, which in turn impairs endothelium-dependent vasodilation [97]. ROS, particularly O_2_^•−^, bind NO and form highly reactive and dangerous ONOO^−^. This ONOO^−^ produces a cascade of changes, which in turn lead to increased tension within the blood vessels.

Lipoic acid may have a beneficial effect in preventing the development of hypertension by lowering the level of inflammatory cytokines in the blood plasma, thus preventing these pathological changes to vessel cells and normalizing changes in blood pressure [98, 99]. Several clinical trials have shown that LA inhibits the vascular overproduction of endothelin I, the main vasoconstrictor [100]. Furthermore, LA significantly increases the synthesis of NO, the main vasodilator; it may also improve the redox state of the plasma and improve endothelium-dependent NO-mediated vasodilation. In addition, LA ameliorates the loss of eNOS phosphorylation, which contributes to improved endothelial function [101, 102]. It is also known to inhibit TNF-alpha activation [103]. As LA is a good metal chelator, it may also inhibit the production of adhesion molecules by monocytes, thus improving endothelial function. In one study, LA supplementation was found to reduce the aortic expression of adhesion molecules and proinflammatory factors, such as lowering the accumulation of aortic macrophages [96].

Furthermore, LA could potentially regulate intracellular Ca^2+^ levels by preventing the modification of sulfhydryl groups in the Ca^2+^ channels [104]. Another study shows that LA increases tissue GSH levels, which otherwise decline with age, by restoring glutathione peroxidase activity [105, 106].

The antioxidant properties of LA cause it to exert a “rejuvenative” impact on mitochondria by protecting them against the higher levels of ROS they produce during the aging process. LA increases oxygen consumption and mitochondrial membrane potential, while decreasing the mitochondrial production of oxidants by amplifying the activity of antioxidant mechanisms [107]. However, despite LA supplementation not being particularly effective in this regard, it can nevertheless significantly reduce blood pressure when used in combination with other antioxidants such as L-carnitine [108].

### 3.3. Effect of Lipoic Acid in Atherosclerosis-Ischemic Heart Disease

Ischemia injury can follow oxidative stress and can lead to significant morbidity and mortality. During ischemia, specific changes in the antioxidant system can occur, resulting in injury to organs such as the kidney, liver, or heart.

In ischemia, oxidative stress causes many complication reactions involving adhesion molecules and cytokines, leading to massive release of ROS. This process increases the production of tumor necrosis factor-alpha (TNF-*α*) and interleukin-1 (IL-1) through activation of NF-*κ*B. Furthermore, increases in intracellular Ca^2+^ concentration and MDA levels result in decreases in GPx and SOD reactivity [109], thus inducing contractile dysfunction, hypertrophy, fibrosis, and cell death [110]. Contractile function and arrhythmias may also be depressed [111]. Clinical studies indicate that up to 50% of the final infarct may be attributable to ischemia injury in both animals and humans [67].

LA counteracts the damage associated with the ischemia experimental model. It can provide protection against ischemia by inhibiting ROS production, blocking inflammation, and reducing myocardium apoptosis, as noted above. Recent studies indicate that LA prevents postreperfusion arrhythmias and protects cardiomyocytes from hypoxia-induced death [112]. It induces cardioprotection through a number of routes: inhibition of NOX4 activity leading to NOS recoupling, improved NO bioavailability, and reduced oxidative stress, leading ultimately to the preservation of mitochondrial function. In addition, LA limits further damage caused by ischemia by increasing Akt phosphorylation via the activation of the PI3K/Akt pathway and the induction of cytoprotective genes [113]. It also prevents decreases in ATP content and the activation of proinflammatory factor NF-*κ*B. In animal models of ischemia, LA was found to ameliorate cardiac dysfunction with reduced infarct size and lower levels of myeloperoxidase, TNF-*α*, creatinine kinase, and lactate dehydrogenase, while upregulating the expression of several antioxidant enzyme genes [114]. Other studies report that LA administration bestows significant protective effects by raising MDA levels and lowering the activity of glutathione peroxidase (GPx) and superoxide dismutase (SOD), the enzymatic scavengers of ROS [109].

Another way to protect the cardiovascular system from oxidative stress is based on its capacity to regenerate endogenous antioxidants such as vitamins C and E. It also regenerates glutathione, which plays a very important role in maintaining the balance between antioxidants and prooxidants. LA may increase the levels of glutathione and other natural antioxidants, thus preventing the progression of ischemia [115].

### 3.4. Effect of Lipoic Acid in Heart Failure

Heart failure (HF) may cause severe damage to the heart muscle via myocardial fibrosis, ventricular remodeling, decreased contractility, and increased myocyte apoptosis [14]. Mitochondrial damage is central to the pathophysiology of HF. The mechanism of mitochondrial dysfunction is connected with cellular and mitochondrial damage which impairs the mechanical properties of the heart. In age-related oxidative stress, a reduced supply of energy from the mitochondria necessary for the contractile function of cardiomyocytes is often noted [116]. Therefore, one strategy in treating HF is the stimulation of cardiac systolic function by targeting mitochondrial dysfunction.

Cardiomyocyte function is disturbed in HF, but not irreversibly [117]. The cardiomyocytes respond to oxidative stress by increasing antioxidant system activity: increased thioredoxin system (Trx) activity has been observed, together with greater mRNA expression of several antioxidant enzymes [118]. Myocardial energy efficiency can be improved by up to 30% by using strategies based on increasing glucose oxidation and decreasing fatty acid metabolism [117].

Many studies on animal models have confirmed that LA can prevent progressive remodeling and even improve cardiac function [119]. By acting as a cofactor for enzymatic reactions within the mitochondria, it can improve mitochondrial function by conserving cellular energy [120]. Thus, LA can influence mitochondrial antioxidant status, neutralize ROS, and effectively attenuate mitochondrial damage caused by oxidative stress and the aging process [121]. Antioxidants such as LA are widely regarded as attractive novel agents which can be employed to prevent oxidative stress when targeted at the mitochondria [122]. Several studies have demonstrated that LA administration effectively attenuates cardiac apoptosis [123, 124]. It has been found to attenuate oxidative damage to the mitochondria, with increased GSH levels and enhanced SOD activity being observed [123]. It has also been seen to mediate the elevation of cellular defense, which may be associated with greater resistance to ROS-elicited cardiac cell injury [124]. Finally, it has also been demonstrated that LA reinforces cellular defenses by inducing endogenous antioxidants and phase 2 enzymes in cultured cardiac cells. These have been associated with markedly increased resistance to ROS-elicited cardiomyocyte injury [124].

All the above examples indicate that LA may be helpful in treating HF caused by oxidative stress. It offers a number of benefits concerned with preventing oxidative damage to the mitochondria, on both molecular and genetic levels, even when applied at low concentrations. Its use in this regard merits further study.

## 4. Summary

In the last years, investigations in human and animal models have provided abundant evidence that age-dependent oxidative stress plays an important role in cardiovascular diseases. Studies indicate that antioxidants prevent development of many cardiovascular diseases and may even improve course of diseases, such as atherosclerosis, hypertension, ischemia-reperfusion, or heart failure. It is therefore disappointing that very few applications have been found for antioxidants in these diseases.

Lipoic acid can provide protection against ROS-induced damage under conditions of elevated oxidative stress brought on by the aging organism. It meets all the criteria for an ideal antioxidant, because it may reduce adverse effects of oxidative stress, has amphiphilic properties, and does not exhibit any serious side effects [125].

However, the results of clinical trials intake of exogenous antioxidant are contradictory. No beneficial effects were reported in several studies in which only one synthetic antioxidant was used. Therefore, a better antioxidant effect can be achieved using more than one antioxidant.

## Abbreviations

ROS: Reactive oxygen species
LA: Lipoic acid
NO: Nitric oxide
RNS: Reactive nitrogen species
NOS: Nitric oxide synthase
CVD: Cardiovascular disease
ox-LDL: Oxidized low-density lipoprotein
TNF-*α*: Tumor necrosis factor-alpha
ET-1: Endothelin-1
AT II: Angiotensin II
NAD(P)H: Nicotinamide adenine dinucleotide phosphate
RAAS: Renin-angiotensin aldosterone system
ATP: Adenosine 5′-triphosphate
HF: Heart failure
mtDNA: Mitochondrial DNA
GSH: Reduced glutathione
SOD: Superoxide dismutase.
